# One Platform, a Thousand Worlds: On Twitter Irony in the Early Response to the COVID-19 Pandemic in Italy

**DOI:** 10.1177/2056305120948254

**Published:** 2020-07-30

**Authors:** Stefania Vicari, Maria Francesca Murru

**Affiliations:** 1 The University of Sheffield, UK; 2 The University of Bergamo, Italy

**Keywords:** COVID-19, Twitter, irony, digital methods, platform studie

## Abstract

On 22 February 2020, 11 municipalities in Northern Italy became the first COVID-19 red zone of Europe. Two days later, when it became evident that the virus had been spreading in the country for weeks, Italy entered a “buffer zone,” a temporal zone between normality and pandemic. The buffer zone lasted around 2 weeks and thrived with irony flowing on social media through memes, multimedia remixes, and jokes. As a collective ritual, irony allowed people to temporarily background the mounting feelings of bewilderment and uncertainty by foregrounding the familiar scripts of playful and grassroots expressivity typical of networked publics. While giving the country a way to breathe before grieving, irony delivered both traditional political satire and new symbolic arrangements to frame “us” versus “them”: Northern Italy versus Southern Italy, Italy versus China. We advance initial reflections on irony and its functions during what we call Italy’s COVID-19 buffer zone and argue for the need of more platform research interested in how users appropriate devices and vernaculars in ways that are culturally bound. In other words, can we rethink “The Platform” (e.g., Twitter, Facebook, Instagram) as a constellation of small-world-platforms—sometimes overlapping, other times segregating—each shaped by local hopes and fears, histories and events?

On 22 February 2020, 11 municipalities in Northern Italy became the first COVID-19 red zone of Europe. Among these was Codogno, the village in Lombardy where “patient 1” of the Italian outbreak was found. Two days later, when it became evident that COVID-19 had been spreading undetected for weeks, Italy entered a “buffer zone,” a temporal zone between normality and pandemic. The buffer zone lasted around 2 weeks and thrived with irony flowing on social media through memes, multimedia remixes, and jokes. With the news reporting of COVID-19 patients around the world who had recently been in Codogno and of people fleeing the Italian red zone, Twitter comments were of the sort of:

We’ll soon learn that Wuhan patient zero was actually from Codogno^1^

On 28 February, Luca Zaia, President of Veneto region commented, “I think that China has paid a big price for this epidemic because we have all seen them eat live mice or things like that” (Reuters, 2020). Italian Twitter did not take long to hit back:

I die for rat roasts

Well, if people from Veneto eat cats, someone needs to take care of mice

We have all seen them—the Vicentini—eat live cats

#Zaia says sorry to the Chinese about #livemice. He swears on his cats

You should not be surprised if these messages do not sound extremely amusing to you. Irony is embedded in culture. If you are not an Italian born in or before the 1980s, in the first tweet, you are unlikely to catch a reference to a cult parody movie from the 1980s where the protagonist—the unlucky Italian clerk Ugo Fantozzi—is offered a “rat fish” roast. If you are not Italian, you are also unlikely to smile at the remaining messages as you probably do not know that people from the city of Vicenza (i.e., “Vicentini”) in Veneto, are traditionally called “magnagati,” that is, “cat eaters” (see Wikipedia, 2020).

And yes, Zaia apologized a day later.

## One Platform, a Thousand Worlds

Irony is an affective rhetorical device able to connect or disconnect publics. In its saying something while meaning something else, the ironic text provides both bonding fabric and boundary work; it bonds those who get it and alienates those who cannot. In a successful ironic exchange, a sort of “conspiratorial pleasure” (Friedman & Kuipers, 2013)—a form of intimacy—builds between the addresser and the addressee. The derived affective bond, based on shared sets of knowledge, norms, conventions, and contexts, translates into a shared sense of belonging that, when missing, puts addresser and addressee on the opposite sides of a boundary (Gal, 2019).

Given that most mainstream social media platforms are designed to nurture, datafy, and quantify public affect in the form of public emotions (Wahl-Jorgensen, 2019, p. 165), it is not surprising that social media discursive sociality has a strong affective dimension (Papacharissi, 2015) where irony can fit extremely well. In fact, in its live streaming, multimodal and polyvocal architecture, Twitter is particularly conducive to irony: tweets are short, fast, and multimedia so it “only” takes a bit of wit to turn them into puns. And yet, irony is not an affective device that can be easily datafied or quantified—it does not directly deliver the kind of affective leaning (e.g., positive or negative evaluation) that you can quickly and massively detect through platform metrics or specific automated sentiment analysis techniques. It does take the eye of an insider to find the clues to unlock irony’s hidden meaning. In other words, to investigate platform irony, we need to take into account socio-cultural cues, medium features (e.g., platform vernaculars), and content (Gal, 2019).

Acknowledging that social media host cultural nuance so very grounded in language and space, like we see in the ironic exchanges in the Italian COVID-19 buffer zone, requires us to reflect on our means and methods to study platforms and platform practices in relation to global themes. As a matter of fact, due to ethical challenges and application programming interface (API)-constrained data access (Walker et al., 2019), platforms are increasingly investigated in relation to global events (e.g., the COVID-19 pandemic) or globally contested causes (e.g., climate change). Amid this progressive shift of focus toward issue-based social media practices, a number of scholars have been calling for cross-platform approaches to truly grasp cultures of use (Pearce et al., 2020; Rogers, 2019, p. 221).

But is cross-platform analysis enough? Are platform devices and vernaculars the most important key to understand user cultures? Should we not constantly try to understand how users appropriate these devices and vernaculars in ways that are culturally bound (Brock, 2012)? And then, during global phenomena, can we look at social media as countries’—or, even better, cultures and subcultures’—affective mirrors? Ultimately, can we rethink “The Platform” (i.e., Twitter, Facebook, Instagram) as a *constellation of small-world-platforms*—sometimes overlapping, other times segregating—each shaped by local hopes and fears, histories and events?

## Twitter Irony During the COVID-19 Buffer Zone in Italy

As overwhelming and unexpected events, epidemics are watersheds that break routines and undermine the taken for granted. Their outbreak is accompanied by a variably long phase of transition during which the most common interpretive frames gradually lose their presumed ability to decode reality and steer paths of actions.

Media have always played a significant role during epidemics, especially by amplifying the pressing quest for new social meanings and by drafting provisional responses to it. They spread useful information and compose new narratives through which contagion is normalized through the use of recognizable plots (Wald, 2008). Media also offer multifaceted mirrors where the emotional wave generated by the unexpected can be recognized and represented. Twitter conversations fit very well in this role, inasmuch as they offer an updated repository of last-minute news and a rhythmic structure for collaborative storytelling (Papacharissi, 2016).

But what is the social function of the copious stream of humor that flooded the Italian Twitter-sphere after “patient 1” was found?

It is not news that tragedy brings comedy with itself. When life becomes hard, irony is a tactic of displacement that lightens bad feelings and frames them from a detached perspective. However, when it takes place on social media, humor can be remediated not only in its expressive forms but also in its social function (Couldry & Hepp, 2013).

If we look at the varied forms of irony during the Italian COVID-19 buffer zone, we can find a recurring theme that has to do with the domestication of the unprecedented. “Amuchina”—a common disinfectant indicated by public health authorities as one of the crucial weapons against contagion—gave rise to a long chain of parodies of popular sitcom and movie titles like “How I infected your mother” (from “How I met your mother”) or “Last Flacon in Paris” (from “Last Tango in Paris”).

In these examples, irony springs from the jarring association of the taken for granted of the past (e.g., cult movies and sitcoms) with elements becoming essential in the present (e.g., Amuchina). More than a mere relief, this kind of irony materialized a collective attempt to manage the mounting feeling of astonishment and the reiterated oscillation between belief and disbelief experienced during the buffer zone. While the main interpretive frames were failing, puns mitigated the solitary exit from the comfort zone by providing individuals with a public and playful recognition of being in the same boat.

But do these dynamics tell us something more about the way Twitter mirrored the country’s “feelings” during the buffer zone? The Italian case suggests that Twitter had a peculiar way to weave together public expressions of irony and underlying emotions. Emotions came to be processed through rituals that reverberated from the individual to a symbolic space of communality. This can be more clearly understood if we think of social media irony first as a practice and then as a content.

As a cumulative and reiterated practice, irony on Twitter is generally characterized by a ritual dimension consisting of scripts performed within a communal context. Like other social rituals, irony is an occasion for personal expression and social interaction that serve solidary functions for both individuals and communities (Yang & Jiang, 2015). As shown in the chain of tweets presented earlier, in the Italian COVID-19 buffer zone, the acts of producing, commenting, sharing, and liking jokes, memes and multimedia remixes stood out as public signs of participation in a ritual that was attended by single individuals but had collective meaning.

**Figure 1.**
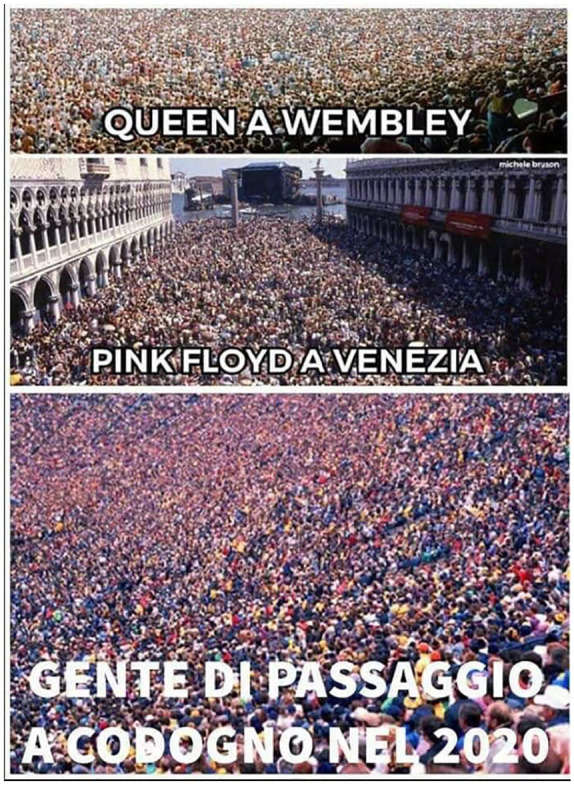
Codogno meme circulating in the buffer zone. Translation: “Queen in Wembley; Pink Floyd in Venice; People passing through Codogno in 2020.”

As content, irony provides the perfect symbolic fabric to highlight sudden transformations in the binary oppositions that structure social life (e.g., safe/unsafe, legal/illegal, lay/expert, close/far). In Italy, the COVID-19 pandemic redefined the social geography of distance and proximity. With the lockdown being gradually intensified and extended to the entire country, Italians realized that China was closer than ever, while relatives and friends living a few miles away had suddenly become unreachable. Irony on Twitter perfectly pictured these paradoxes. The names of places came to be re-written with a resonance to China: Sesto San Giovanni, a city in the Milan’s hinterland became “Sesto San Wuhan.”^2^ The official reconstruction of the spatial evolution of the epidemic as departing from Codogno was mocked with puns underlining the sudden coming to global prominence of the previously unknown little village (see Figure 1).

**Figure 2.**
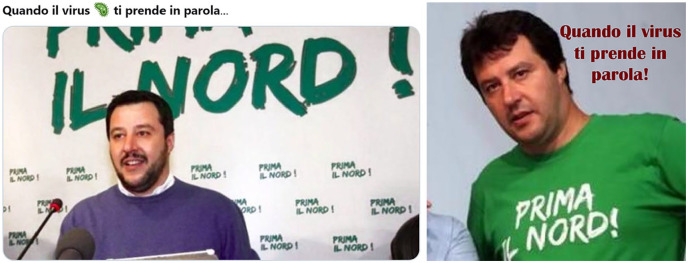
Two versions of the “Prima il Nord!” (“North First”) meme. Translation: “When the virus takes you at your words.”

The uneven spread of the virus across the country, with a strong prevalence in the richer regions of the North, renovated the long-standing conflictual opposition North versus South. A variety of pictures reporting the political campaign slogan “North first” alongside the figure of Matteo Salvini—candidate for the once secessionist Northern League party—circulated with the comment “When the virus takes you at your words” (Figure 2).

In its relying on known scripts (e.g., virality, memetics, multimodality), Twitter irony shuffled some of Italy’s traditional divides, uncovering ancestral wounds (e.g., North-South antagonism), while incorporating COVID-19 uncertainties in the country’s cultural fabric. It symbolically evoked a communal space where emotions could be pulled out of the private sphere and publicly shared. As a playful reaction to many of the symbolic ruptures caused by the pandemic, it embodied the country’s awareness of the ongoing transformations affecting individual and social lives. Ultimately, it triggered a sort of collective intelligence through which the unexpected was domesticated and the mounting feeling of astonishment recognized.

## Platforms as Culturally Bound

Hopefully, you will now have a sense of what Italian Twitter looked like in late February–early March 2020. If anything, this reflection shows us that the platform mirrored Italy’s atavic divides and contextual fears, bringing them together in new collective affective structures.

But platforms—and user cultures—are far from monolithic. It is only plausible that in the same period Twitter uses, practices and content looked and functioned extremely differently in the remaining COVID-19 affected world. Twitter was definitely something else, elsewhere.

But then, if we aim to better understand platforms’ roles in relation to global issues, should we not try to advance more cross-cultural platform studies? Is it not only by exploring platforms as culturally bound—as located in place, time, and context—more than as unbending entities, that we can truly grasp their roles, functions, and dysfunctions in societ*ies*?

## Notes

1. All tweets reported in this article were translated by the authors.

2. The naming was generally used without a xenophobic dimension.
